# Gut Microbiota Modulate Rabbit Meat Quality in Response to Dietary Fiber

**DOI:** 10.3389/fnut.2022.849429

**Published:** 2022-03-22

**Authors:** Boshuai Liu, Yalei Cui, Qasim Ali, Xiaoyan Zhu, Defeng Li, Sen Ma, Zhichang Wang, Chengzhang Wang, Yinghua Shi

**Affiliations:** ^1^The College of Animal Science and Technology, Henan Agricultural University, Zhengzhou, China; ^2^Henan Key Laboratory of Innovation and Utilization of Grassland Resources, Zhengzhou, China; ^3^Henan Forage Engineering Technology Research Center, Zhengzhou, China

**Keywords:** rabbit, gut microbiota, dietary fiber, meat quality, metabonomics

## Abstract

Antibiotics are widely used in gastrointestinal diseases in meat rabbit breeding, which causes safety problems for meat products. Dietary fiber can regulate the gut microbiota of meat rabbits, but the mechanism of improving meat quality is largely unknown. The objective of this study was to evaluate the effects of adding different fiber sources to rabbit diets on the growth performance, gut microbiota composition, and muscle metabolite composition of meat rabbits. A total of 18 New Zealand white rabbits of similar weight (40 ± 1 day old) were randomly assigned to beet pulp treatment (BP), alfalfa meal treatment (AM), and peanut vine treatment (PV). There were 6 repeats in each treatment and all were raised in a single cage. The predictive period was 7 days and the experimental period was 40 days. The results revealed that AM and PV supplementation increased growth performance, slaughter performance, and intestinal development of meat rabbits compared with the BP treatment, and especially the effect of AM treatment was better. The content of butyric acid was increased in PV and AM treatments compared with the BP treatment. The expression of mitochondrial biosynthesis genes of liver, cecum, and muscle showed that AM treatment increased gene expression of CPT1b compared to the BP treatment. In addition, AM and PV treatments significantly increased the microbial diversity and richness compared with BP treatment, and their bacterial community composition was similar, and there were some differences between AM and PV treatments and BP treatment. Metabonomics analysis of muscle showed that AM treatment significantly increased amino acid and fatty acid metabolites compared with BP treatment, which were mainly concentrated in energy metabolism, amino acid metabolism, and fatty acid regulation pathways. Furthermore, through correlation analysis, it was found that there was a significant correlation between rumenococci in the cecum and amino acid metabolites in the muscle. Overall, these findings indicate that AM may affect the body's health by changing its gut microbiota, and then improving meat quality, and the intestinal–muscle axis provides a theoretical basis.

## Introduction

With the transformation of the meat rabbit breeding industry from free-range to large-scale production and the universal application of pellet feed, the phenomenon of gastrointestinal diseases and poor meat quality of meat rabbits has caused great losses to meat rabbit production (1–3). In the past, antibiotics were used to prevent and treat these diseases, but owing to broader application and abuse of antibiotics may cause increasingly serious issues such as bacterial drug resistance genes and meat product safety. Therefore, there is an urgent need for a method to solve the above problems (4, 5). Meat rabbits are monogastric herbivores. Adequate dietary fiber supply can prevent diarrhea in meat rabbits, which has been confirmed by relevant studies (6). Therefore, the application of feed restriction is an efficient alternative to reduce the use of antimicrobials to prevent the onset of enteric diseases.

Rabbit is a single stomach herbivore. Compared with other single stomach animals, the intestinal structure of rabbits is relatively unique. The cecum volume of rabbits can reach about 49% of the total volume of all gastrointestinal tracts, which is the largest among single stomach animals (7). The cecum of rabbits is the main fermentation organ with abundant and diverse bacteria that help them to break down indigestible substances (8). Rabbits do not secrete cellulolytic enzymes. Therefore, in the process of digesting feed, the utilization and digestion of dietary fiber mainly depend on the bacteria growing in the cecum of rabbits to ferment dietary fiber into short-chain fatty acids and other substances by phagocytosis and secretion of cellulolytic enzymes (9). This process not only stimulates intestinal development but also optimizes gut microbiota and intestinal barrier function and even can affect the metabolism of the liver, brain, and muscle tissue, and thus help to accentuate the host's overall nutrient metabolism network, animal behavior, and cognitive level (10, 11). Therefore, the nutritional strategy of regulating the fermentation of rabbit cecum through dietary fiber can ensure the healthy development of rabbits (12–14).

The muscle quality of meat rabbits is considered to be the key growth characteristic of meat rabbits (15). However, a few studies have revealed the relationship between gut microbiota and rabbit meat quality, which may affect muscle metabolism. For example, after transplanting bacteria from obese or lean pigs, sterile mice replicated the fiber characteristics and lipid metabolism characteristics of donor skeletal muscle (16). Interestingly, supplementation of probiotics in mice can also improve muscle quality, promote the slow oxidative muscle phenotype associated with increased muscle endurance, and reduce exercise-induced muscle injury (17). When the gut microbiota is destroyed, the availability of energy substrate is reduced, especially the reduction of glucose may lead to the reduction of muscle glycogen storage to maintain glucose homeostasis, which could prone to muscle injury (18). Therefore, gut microbiota may regulate meat quality through energy metabolism. cAMP–PKA–AMPK signaling pathway is an important energy sensor and regulator in cell life activities (19). It is found that NRF1 and CPTlb are enriched in tissues with high oxidation capacities, such as brown adipose tissue, brain, heart, and skeletal muscle (20). The correlation analysis between gut microbiota and metabolome can better explain the mechanism of physiological activities.

At present, the research on rabbit feed nutrition is at the level of crude fiber. Due to the wide source, variety, and complex chemical structure of the dietary fiber, the functions and effects of different dietary fibers are very different (3, 21). Under the condition of appropriate fiber level in the meat rabbit diet, different fiber types should have a certain impact on the growth of meat rabbits, and it is particularly important to select the appropriate fiber types. In addition, systematic evaluation of different fiber sources (mainly including insoluble fiber sources and soluble fiber sources) has less research on gut microbiota and muscle metabolic composition of meat rabbits. Therefore, in this study, we evaluated the effects of adding different fiber sources, including beet pulp (BP), alfalfa meal (AM), and peanut vine (PV), to rabbit diets on the growth performance, gut microbiota composition, and muscle metabolite composition of meat rabbits, to provide a scientific theoretical basis for the use of different fiber sources in the daily feed preparation of meat rabbits.

## Materials and Methods

### Animals, Diets, and Housing

A total of 18 New Zealand white rabbits of similar weight and age (1,478.43 ± 178.31 g, 40 ± 1 day of age) were randomly assigned to BP treatment, AM treatment, and PV treatment. There were 6 repeats in each treatment and all were raised in a single cage. The predictive period was 7 days and the experimental period was 40 days. The diets of growing and fattening rabbits were formulated according to the NRC (2012). The composition and nutritional composition of diets are shown in Supplementary Table S1. The diets of each treatment were processed into pellets and fed two times a day, that is, at 06:00 and 18:00 each. Free drinking and conventional feeding management were used during the trial, and environmental conditions were consistent. The rabbit shed was cleaned daily, disinfected one time in a week, was provided with natural lighting and ventilation, and the rabbit shed temperature was maintained at 15–25°C. Individual body weight (BW) was recorded on Day 1, Day 7, and Day 47, and feed consumption was recorded daily to determine the average daily feed intake (ADFI), average daily weight gain (ADG), and F/G.

### Sample Collection

At the end of the experiment, 6 New Zealand white rabbits were sampled for each treatment. The gastrointestinal tract of each meat rabbit was taken out immediately after slaughter, and the middle part (2 cm) of duodenum, jejunum, and ileum was collected and fixed in 10% formaldehyde buffer for intestinal morphology analysis. Liver, cecal, and longissimus dorsi muscle segments were collected and immediately frozen in liquid nitrogen for real-time quantitative PCR. Chyme samples of cecum were collected, and cecal mucosa was washed with sterile cold phosphate buffer. Samples of cecal mucosa were collected using sterile glass microscope slides. The longissimus dorsi muscle of the meat rabbit was collected. All samples were stored in sterile cryopreservation tubes and immediately frozen at −80°C until further analysis.

### Determination of Slaughter Performance Index

At the end of the test, before slaughter, the meat rabbit was weighed and the live weight was recorded, and the length from neck to tail root was measured with a soft ruler. After jugular vein bloodletting, the skin was immediately stripped, forelimbs were removed at the wrist joint and hind limbs were removed at the tarsal joint, and intestinal tract and contents (keeping head, thoracic organs, liver, kidney, and fat around the kidney) were removed and weighed, that is, semi-clean bore weight. The total carcass weight was calculated by removing the head and all internal organs (only the kidney and the fat around the kidney), and the half carcass slaughter rate and the total carcass slaughter rate are calculated, respectively.

### Determination of Muscle Quality

After the rabbits were slaughtered, the left longissimus dorsi muscle (3 cm × 4 cm in size) was removed with a scalpel to measure the pH and color of the muscle for 24 h. Insert the probe of Mettler MP120 pH meter into the muscle 3 mm at the fifth rib of the longissimus dorsi muscle, which is the muscle pH. CIE-Lab output mode was used to cut three sections from the longissimus dorsalis muscle, and the values of brightness (L^*^), redness (a^*^), and yellowness (b^*^) were recorded.

### Mucosal Morphology Measurements

After the intestinal segments (duodenum, jejunum, and ileum) were fixed in 10% formaldehyde buffer for 24 h, dehydration, clearing, and paraffin embedding were performed. Then serial sections of 5 μm thickness were made, and this was followed by hematoxylin and eosin staining. Two transverse sections of each intestinal sample (duodenum, jejunum, or ileum) were prepared on one slide for morphometric analysis. A total of 15 intact, well-oriented crypt-villus units per sample were chosen randomly and measured. Villus height measurements were taken from the tip to the base of the villus between individual villus, and crypt depth was measured from the valley between individual villi to the basal membrane. The small intestinal crypt depth (μm) and villus height (μm) were measured at 40× magnification with an Olympus CK 40 microscope (Olympus Optical Company), and then villus height/crypt depth (V/C) was calculated as the villus height divided by the crypt depth.

### Gene Expression Analysis by Real-Time Polymerase Chain Reaction

Total RNA was isolated from liver and cecum tissue by using MagPure Universal RNA LQ Kit (R6623; Magen Biotechnology Co., Ltd., Guangzhou, China) according to the manufacturer's instructions. The concentration and purity of RNA samples were determined on a NanoDrop ND-1000 spectrophotometer (Thermo Scientific, Waltham, MA, United States). Single-stranded cDNA was synthesized from 1 μg of total RNA by using the PrimeScript RT kit (Toyobo) (FSQ-301; TOYOBO, Tokyo, Japan) according to the manufacturer's instructions. Quantitative real-time PCR was carried out with the use of ChamQ Universal SYBR qPCR Master Mix (Q711-02; Vazyme Biotech Co. Ltd., Nanjing, China). The reaction system of quantitative real-time PCR was 10 μL including 5 μL 2 × ChamQ Universal SYBR qPCR Master Mix, 0.5 μL cDNA, 0.2 μL forward and reverse primers (10 mmol/L), and 4.1 μL double-distilled water. Quantitative real-time PCR was performed on LightCycler 96 real-time PCR instrument (Roche). The expression of the genes was calculated relative to the expression of the housekeeping gene β*-actin* with the formula 2^−Δ*ΔCt*^. Amplification of specific transcripts was confirmed by melting curve profiles at the end of each PCR. The primer sequences are listed in Supplementary Table S2.

### Determination of SCFAs Levels

According to Liu et al., gas chromatography (GC) was used to determine the level of short-chain fatty acids (SCFAs) in stool samples. The samples were analyzed on an HP-88 column (100 m long, 0.25 mm diameter, 0.2 film thickness m from the producer) and separated using a TRACE 1310 GC with a flame ionization detector (FID). The programmed temperature was 70°C for 1 min, then increased to 180°C held at 25°C/min and 1 min, then increased to 200°C held at 10°C/min and 1 min, then increased to 220°C held at 2°C/min and 10 min, and finally raised to 240°C held at 20°C/min and 6 min. The sample was run with a split ratio of 20:1 and a column flow of 1.3 ml/min. Hydrogen is used as a carrier gas. The injector temperature was 270°C and the detector temperature was 290°C.

### DNA Extraction and 16S RRNA Gene Sequencing

Microbial DNA was extracted from feces samples using an E.Z.N.A. soil DNA Kit (Omega Bio-Tek, Norcross, GA, United States), according to the manufacturer's protocols. The final DNA concentration and purity were determined using a NanoDrop 2000 UV–vis spectrophotometer (Thermo Scientific, Wilmington, United States), and DNA quality was checked by 1% agarose gel electrophoresis. The V3–V4 hypervariable regions of the bacterial 16S rRNA gene were amplified using the primers 338F (5′-ACTCCTACGGGAGGCAGCAG-3′) and 806R (5′-GGACTACHVGGGTWTCTAAT-3′) by PCR (GeneAmp 9700, ABI, United States), with the following program: 3 min denaturation at 95°C; 27 cycles of 30 s at 95°C, 30 s annealing at 55°C, and 45 s elongation at 72°C; and a final extension at 72°C for 10 min. PCR reactions were performed in triplicate, with each 20 μL reaction mixture containing 4 μL of 5 × FastPfu Buffer, 2 μL of 2.5 mM dNTPs, 0.8 μL of each primer (5 μM), 0.4 μL FastPfu Polymerase, and 10 ng template DNA. The resulting PCR products were extracted from 2% agarose gels, further purified using the AxyPrep DNA Gel Extraction Kit (Axygen Biosciences, Union City, CA, United States), and quantified using a QuantiFluor-ST instrument (Promega, United States), according to the manufacturer's protocol. Purified amplicons were pooled in equimolar amounts and subjected to paired-end sequencing (2 × 300 bp), on an Illumina MiSeq platform (Illumina, San Diego, United States) according to standard protocols, by Majorbio Bio-Pharm Technology Co. Ltd. (Shanghai, China).

### Bioinformatics Analysis of Sequencing Data

Raw fastq files were demultiplexed, quality-filtered using Trimmomatic, and merged using FLASH, with the following criteria: (i) reads were truncated at any site receiving an average quality score <20 over a 50-bp sliding window; (ii) primers were exactly matched, allowing 2 nucleotide mismatching, and reads containing ambiguous bases removed; and (iii) sequences whose overlap was longer than 10 bp were merged, according to their overlap sequence. Operational taxonomic units (OTUs) were clustered with a 97% similarity cutoff, using UPARSE (version 7.1 http://drive5.com/uparse/), and chimeric sequences identified and removed using UCHIME. The taxonomy of each 16S rRNA gene sequence was analyzed using the RDP Classifier algorithm (http://rdp.cme.msu.edu/) against the Silva (SSU128) 16S rRNA database, with a 70% confidence threshold. All samples have been refined to the same library sample size. Alpha diversity (Sobs, Shannon, and Chao1 richness indices) was calculated based on the OTU profiles from the MOTHUR program (version v.1.30.2 https://mothur.org/wiki/calculators/). Then, the Wilcoxon rank-sum test method was used to detect whether the index value between each of the two groups had a significant difference. The Kruskal–Wallis rank-sum test method was used to identify differences between groups. Beta-diversity measures dependent on Bray–Curtis and unweighted-UniFrac distance were calculated using MOTHUR (Stats package of R (version 3.3.1) and SciPy package of Python).

### Metabolite Extraction and Analysis

After thawing the muscle samples slowly at 4°C, take 50 mg sample and put it into a 2-ml centrifuge tube. Add 500 μL extract (methanol: water = 4:1) and 20 μl internal standard (0.3 mg/ml, L-2-chloro-phenylalanine, prepared with methanol) into the sample. Add a steel ball and crush it with a high-throughput tissue crusher at low temperature (50 Hz, 3 min); 200 μL chloroform was added and broken by high-throughput tissue crusher at low temperature (50 Hz, 3 min). After vortex mixing, ice water bath ultrasonic extraction is to be done for 10 × 3 min. Place the sample at −20°C for 30 min, centrifugation for 20 min (12,000 RCF, 4°C), take the supernatant, put it into a glass derivatization bottle and dry it under vacuum. Add 80 μl of pyridine methoxyamine hydrochloride solution (15 mg/ml) to the glass derivatization vial. After vortex shaking for 2 min, oximation reaction is carried out in the shaking incubator at 37°C for 90 min. After taking out the sample, add 80 μl BSTFA (including 1% TMCs) derivatization reagent and 20 μl *n*-hexane. After vortex shaking for 2 min, react at 70°C for 60 min. After taking out the sample, place it at room temperature for 30 min for GC–MS metabonomics analysis. Muscle metabonomics was entrusted to Shanghai Meiji Biology Co., Ltd.

### Statistical Analysis

Statistical analyses were performed using SPSS 20.0 software (IBM, New York, NY, United States). Data were evaluated by one-way ANOVA, and the differences between the means were assessed using Duncan's test. *P* < 0.05 was considered as statistically significant.

## Results

### Growth Performance and Apparent Digestibility of Meat Rabbits

**Figure 1:**
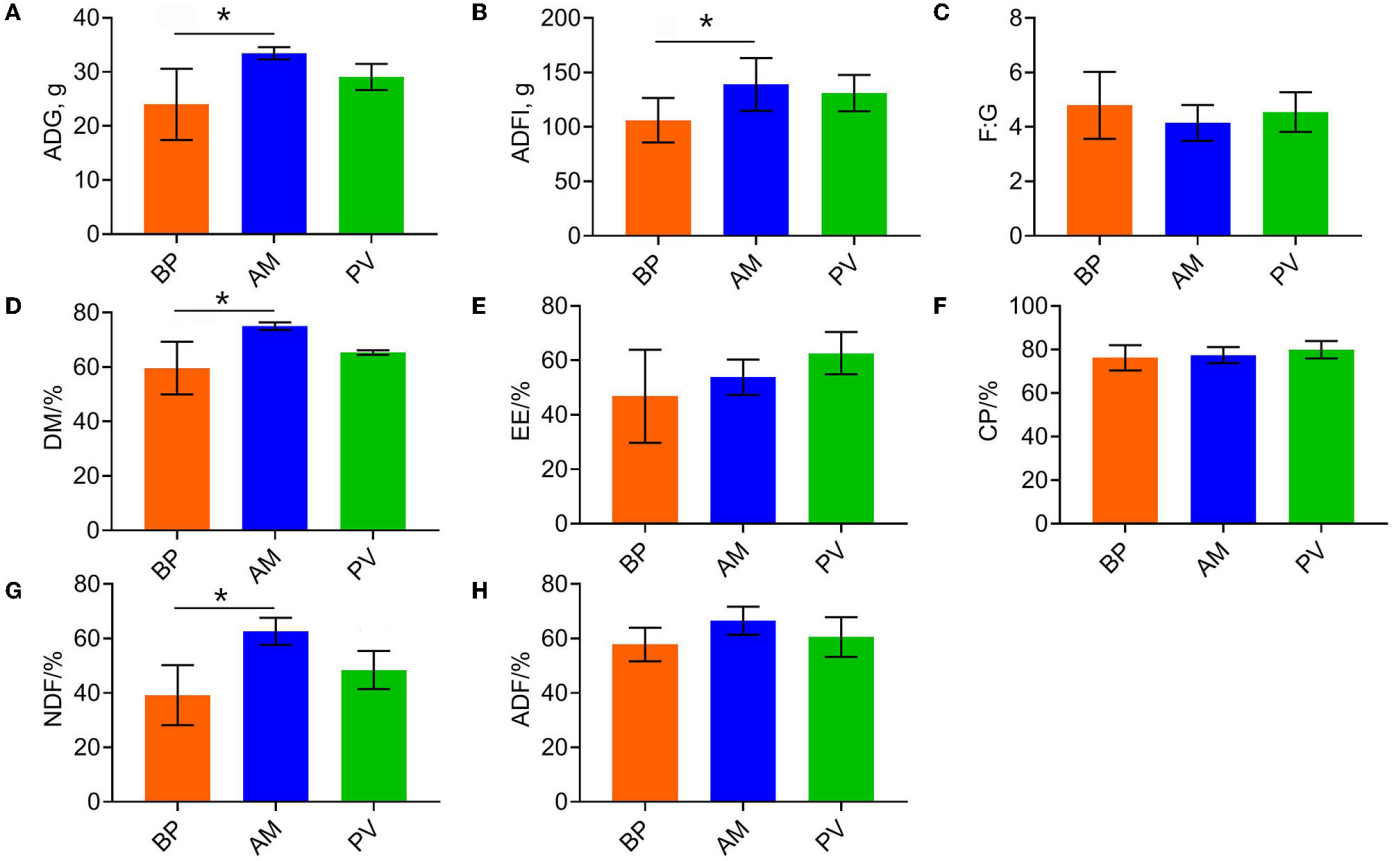
Growth performance and apparent digestibility of meat rabbits. **(A)** ADG. **(B)** ADFI. **(C)** F:G. **(D)** DM. **(E)** EE. **(F)** CP. **(G)** NDF. **(H)** ADF. Average daily gain (ADG), Average daily feed intake (ADFI), Average daily gain: Average daily feed intake (F:G), Dry matter (DM), Ether extract (EE), Crude protein (CP), Neutral detergent fiber (NDF), Acid detergent fiber (ADF), beet pulp treatment (BP), alfalfa meal treatment (AM), and peanut vine treatment (PV). The asterisks symbol indicates significant differences (*0.01 < *P* ≤ 0.05, **0.001 < *P* ≤ 0.01, ***P ≤ 0.001).

The growth performance and apparent digestibility of meat rabbits fed with different fiber source diets were evaluated (Figure 1). Compared with BP treatment, AM treatment significantly improved the average daily gain (*P* < 0.05) (Figure 1A), average daily feed intake (*P* < 0.05) (Figure 1B), and the apparent digestibility of dry matter and neutral detergent fiber (*P* < 0.05) (Figure 1D), where PV treatment showed an increasing trend (*P* > 0.05). Besides, there was no significant difference in feed meat ratio and apparent digestibility of other nutrients among treatments (*P* > 0.05) (Figures 1E–H).

### Slaughter Performance and Intestinal Development of Meat Rabbit

**Figure 2:**
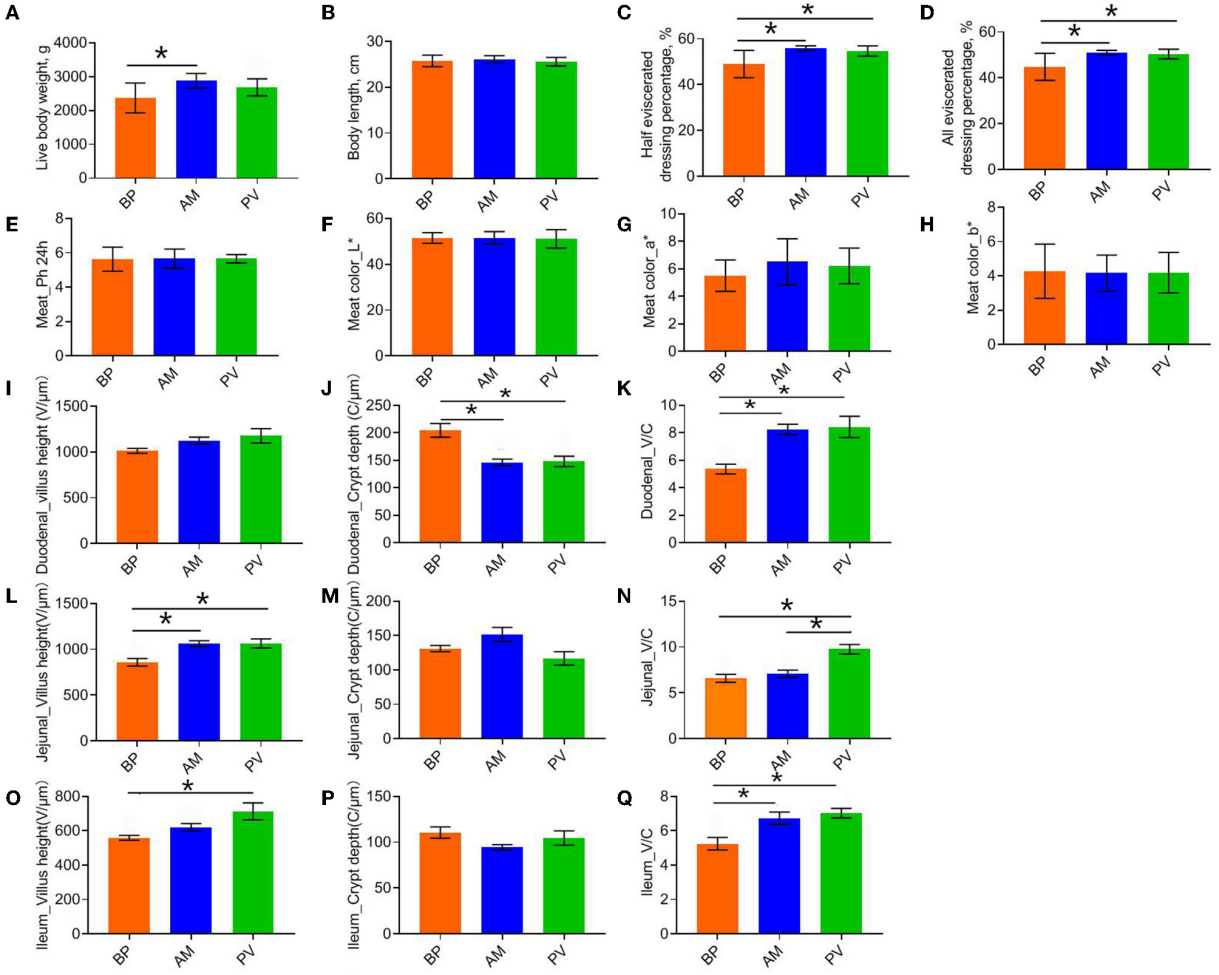
Slaughter performance, foregut development, and cecal fermentation parameters of meat rabbit. **(A)** Live body weight, **(B)** Body lenght, **(C)** Half bore rate, **(D)** Full bore rate, **(E)** Meat_Ph^24h^, **(F)** Meat color_L^*^, **(G)** Meat color_a^*^, **(H)** Meat color_b^*^, **(I)** Duodenal_villus height, and **(J)** Duodenal_crypt depth. **(K)** Duodenal_villus: crypto ratio. **(L)** Jejunum_villus height. **(M)** Jejunum_crypt depth. **(N)** Jejunum_villus: crypto ratio. **(O)** Ileum_villus height. **(P)** Ileum_crypt depth. **(Q)** Ileum_villus: crypto ratio. beet pulp treatment (BP), alfalfa meal treatment (AM), and peanut vine treatment (PV). The asterisks symbol indicates significant differences (*0.01 < *P* ≤ 0.05, **0.001 < *P* ≤ 0.01, ***P ≤ 0.001).

Slaughter performance and intestinal development of rabbits fed with different fiber sources were evaluated (Figure 2). It was found that the premortem live body weight, half and full evisceration rate of AM treatment was higher than those of PV treatment (*P* > 0.05) (Figures 2A,C,D). Compared with BP (*P* < 0.05), AM treatment had no significant difference in body length between treatments (*P* > 0.05). There was no significant difference in muscle sensory quality evaluation (*P* > 0.05) (Figures 2E–H). Further study of intestinal development showed that the crypt depth of AM and PV treatments was significantly lower than that of BP treatment, but the villus:crypto ratio was significantly increased (*P* < 0.05) (Figures 2I–K). In the jejunum, the villus height of PV and AM were significantly higher than those of BP, and the villus:crypto ratio of PV treatment was significantly higher than that of BP and AM treatment (*P* < 0.05) (Figures 2L–N). In the ileum, PV and AM treatments significantly improved compared with BP treatment (*P* < 0.05) (Figures 2O–Q).

### Cecal Fermentation Parameters of Meat Rabbit

**Figure 3:**
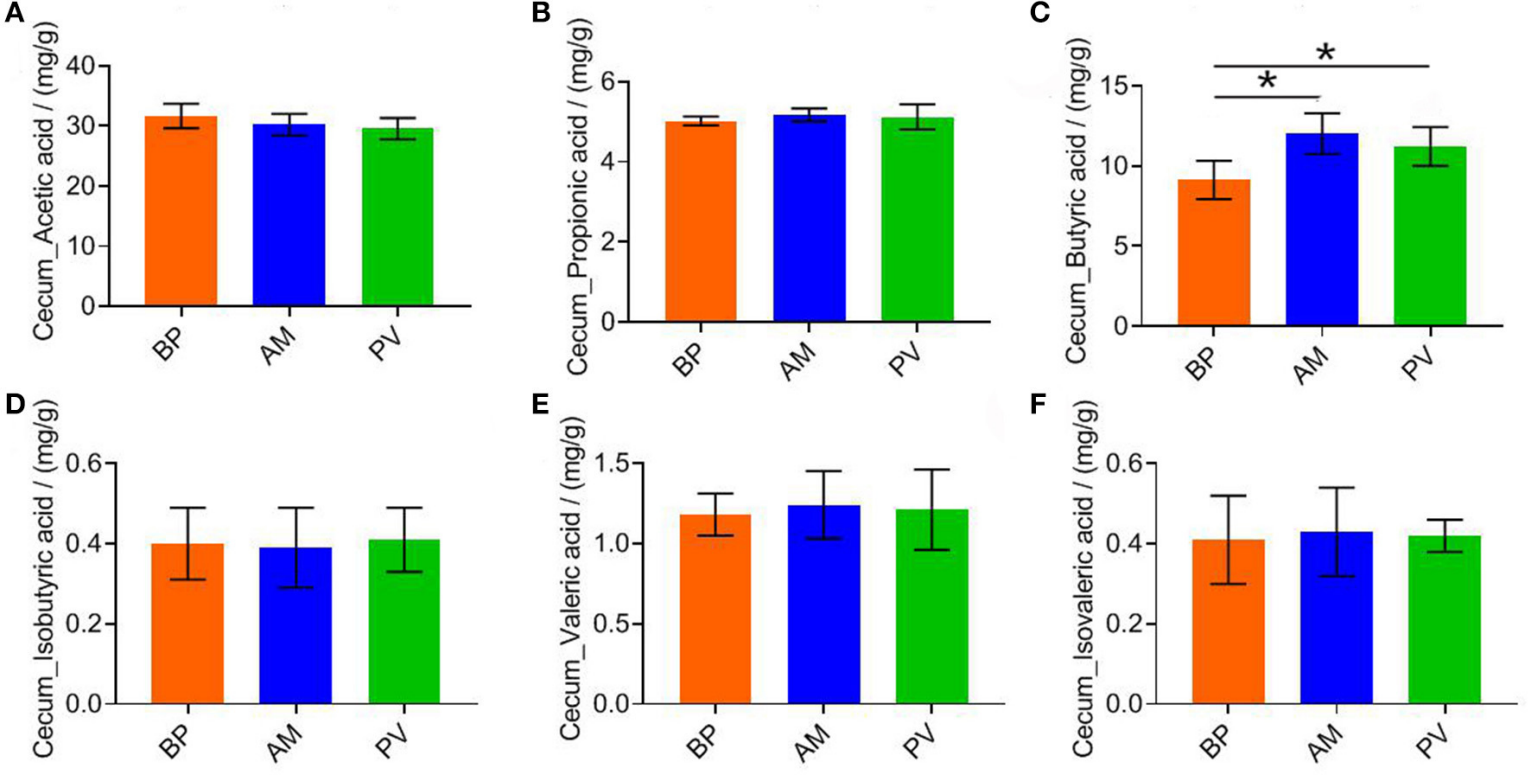
Cecal fermentation parameters of meat rabbit. **(A)** Cecum_Acetic acid. **(B)** Cecum_Propionic acid. **(C)** Cecum_Butyric acid. **(D)** Cecum_Isobutyric acid. **(E)** Cecum_Valeric acid. **(F)** Cecum_Isovaleric acid. beet pulp treatment (BP), alfalfa meal treatment (AM), and peanut vine treatment (PV). The asterisks symbol indicates significant differences (*0.01 < *P* ≤ 0.05, **0.001 < *P* ≤ 0.01, ***P ≤ 0.001).

The cecal SCFAs content of meat rabbits was evaluated after feeding meat rabbits with different fiber source diets (Figure 3), It was found that the content of butyric acid in PV and AM treatments was significantly higher than that in BP treatment (*P* < 0.05) (Figure 3C). There was no significant difference in other SCFAs between treatments (*P* > 0.05) (Figures 3A,B,D–F).

### Expression of Mitochondrial Biosynthesis Genes of Meat Rabbits

**Figure 4:**
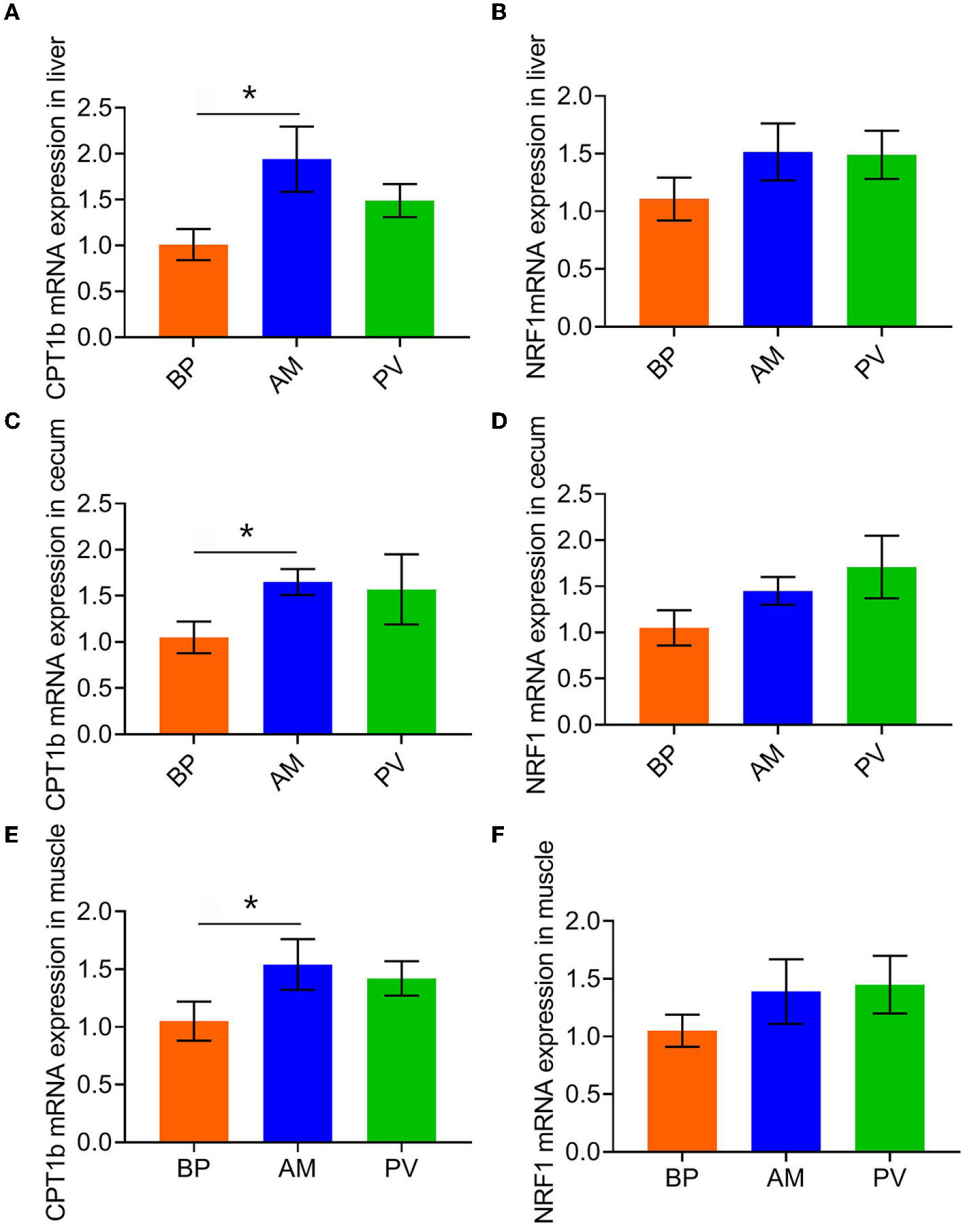
Expression of mitochondrial biosynthesis genes in liver and cecum of meat rabbits. **(A)** CPT1b mRNA expression in liver, **(B)** NRF1 mRNA expression in liver, **(C)** CPT1b mRNA expression in cecum, **(D)** NRF1 mRNA expression in cecum, **(E)** CPT1b mRNA expression in muscle, and **(F)** NRF1 mRNA expression in the muscle. beet pulp treatment (BP), alfalfa meal treatment (AM), and peanut vine treatment (PV). The asterisks symbol indicates significant differences (*0.01 < P ≤ 0.05, **0.001 < *P* ≤ 0.01, ***P ≤ 0.001).

After feeding meat rabbits with different fiber source diets, the expression of mitochondrial biosynthesis genes in liver, cecum, and muscle of meat rabbits was evaluated (Figure 4). It was found that compared with BP, the mRNA expression of CPT1b was significantly higher in AM treatment (*P* < 0.05) (Figures 4A,C,E), and that in PV treatment had a tendency to increase (*P* > 0.05). There was no significant difference in the mRNA expression of NRF1 among treatments (*P* > 0.05) (Figures 4B,D,F).

### Microbiota Community Structure in the Cecum of Meat Rabbits

**Figure 5:**
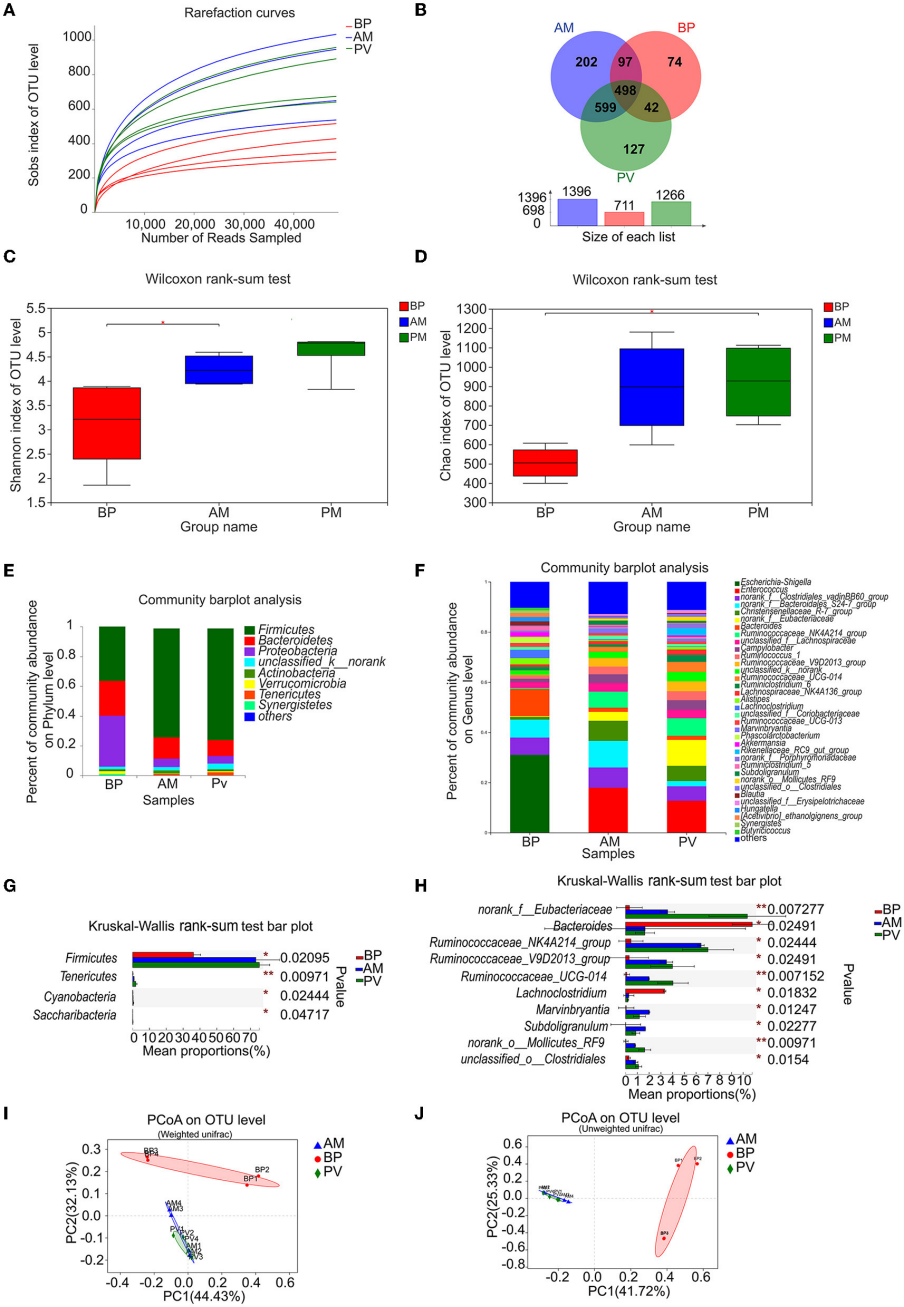
Microbiota community in the cecum of meat rabbits**. (A)** Sobs index of OTU level, **(B)** Venn analysis, **(C)** Shannon index of OTU level, **(D)** Chao index of OTU level, **(E)** Microbiota community at the phyla level, **(F)** Microbiota community at the genus level, **(G)** Composition difference at the phyla level, **(H)** Composition difference at the genus level, **(I)** PCoA on OTU level by weighted-UniFrac distance, and **(J)** PCoA on the OTU level by unweighted-UniFrac distance. beet pulp treatment (BP), alfalfa meal treatment (AM), operational taxonomic unit (OTU), principal coordinates analysis (PCoA), and peanut vine treatment (PV).The asterisks symbol indicates significant differences (*0.01 < *P* ≤ 0.05, **0.001 < *P* ≤ 0.01, ***P ≤ 0.001).

Through the deep sequencing of V3–V4 region of meat rabbit cecum samples fed with different fiber source diets by 16S rRNA gene, the dilution curve tends to be flat, and the sequencing data reach the saturation, which can cover most of the microbiota in the cecum (Figure 5A). A total of 1,639 OTUs were found in the three treatments, including 498 OTUs in three treatments, 599 OTUs between PV treatment and AM treatment, 42 OTUs between PV treatment and BP treatment, and 97 OTUs between AM treatment and BP treatment, indicating that the composition of microbiota in the cecal samples treated with AM and PV is similar (Figure 5B). Further analysis of the abundance and diversity of the microbiota of meat rabbits between different treatments showed that the Shannon index of AM treatment was higher than that of BP treatment (Figure 5C). In addition, the Chao 1 index of PV treatment was significantly higher than that of BP treatment (Figure 5D).

The species composition of bacterial community at the level of phylum and genus in the cecum of meat rabbits with different treatments were presented by a community column diagram. The column diagram shows the proportion of different species in different color areas, where species with abundance below 1% are combined into others (Figures 5E,F). The results showed that *Firmicutes* (73.22%), *Bacteroidetes* (14.54%), and *Proteobacteria* (5.40%) were dominant in AM treatment. The advantage of PV treatment were *Firmicutes* (75.29%), *Bacteroidetes* (10.73%), and *Proteobacteria* (5.19%) were dominant in PV treatment. *Firmicutes* (36.14%), *Bacteroidetes* (23.50%), and *Proteobacteria* (34.46%) were the dominant bacteria in BP treatment. Further study on the genus level showed that *Enterococcus* (17.88%) was the dominant species in AM treatment; *norank_f__Clostridiales_vadinBB60_group* (8.07%), *norank_f__Bacteroidales_S24-7_group* (10.61%), *Christensenellaceae_R-7_group* (8.00%), and *Ruminococcaceae_NK4A214_group* (6.42%). The dominant genera in PV treatment were *Enterococcus* (12.69%), *norank _ f _ Eubacteracea* (10.35%), and *Ruminococcaceae_NK4A214_group* (7.00%), *Christensenellaceae_R-7_group* (6.07%), and *norank_f__Clostridiales_vadinBB60_group* (5.76%). The dominant genera in BP treatment were *Escherichia Shigella* (31.05%), *Bacteroides* (10.77%), and *norank_f__Bacteroidales_S24-7_group* (7.16%), and *norank_f_Clostridiales_vadinBB60_group* (6.86%). The results showed that the composition of bacterial communities in AM and PV samples was similar. Similarly, principal coordinates analysis (PCoA) found that the samples of AM and PV treatments were more similar, and there were certain differences in the ones with BP treatment (Figures 5I,J). Further univariate analysis of variance between the three treatments found that at the phylum level, the relative abundance of *Firmicutes* in AM and PV treatments was significantly higher than that in BP treatment, while that of *Tenericutes* decreased significantly. At the genus level, AM-treated and PV-treated *norank _ f _ Eubacteriaceae, Ruminococcaceae_NK4A214_group, Ruminococcaceae_V9D2013_group*, and the relative abundance of *Ruminococcaceae _ UCG-014* was significantly higher than that of BP, while that of *Bacteroides*, Lachnospiraceae, was significantly lower (Figures 5G,H). There was less difference between AM and PV treatments. Therefore, the above results also showed that the bacterial community composition of AM treatment and PV treatment was similar, and there was some difference between the two treatments and BP treatment.

### Metabolic Spectrum Analysis of Longest Dorsal Muscle in Meat Rabbit

**Figure 6:**
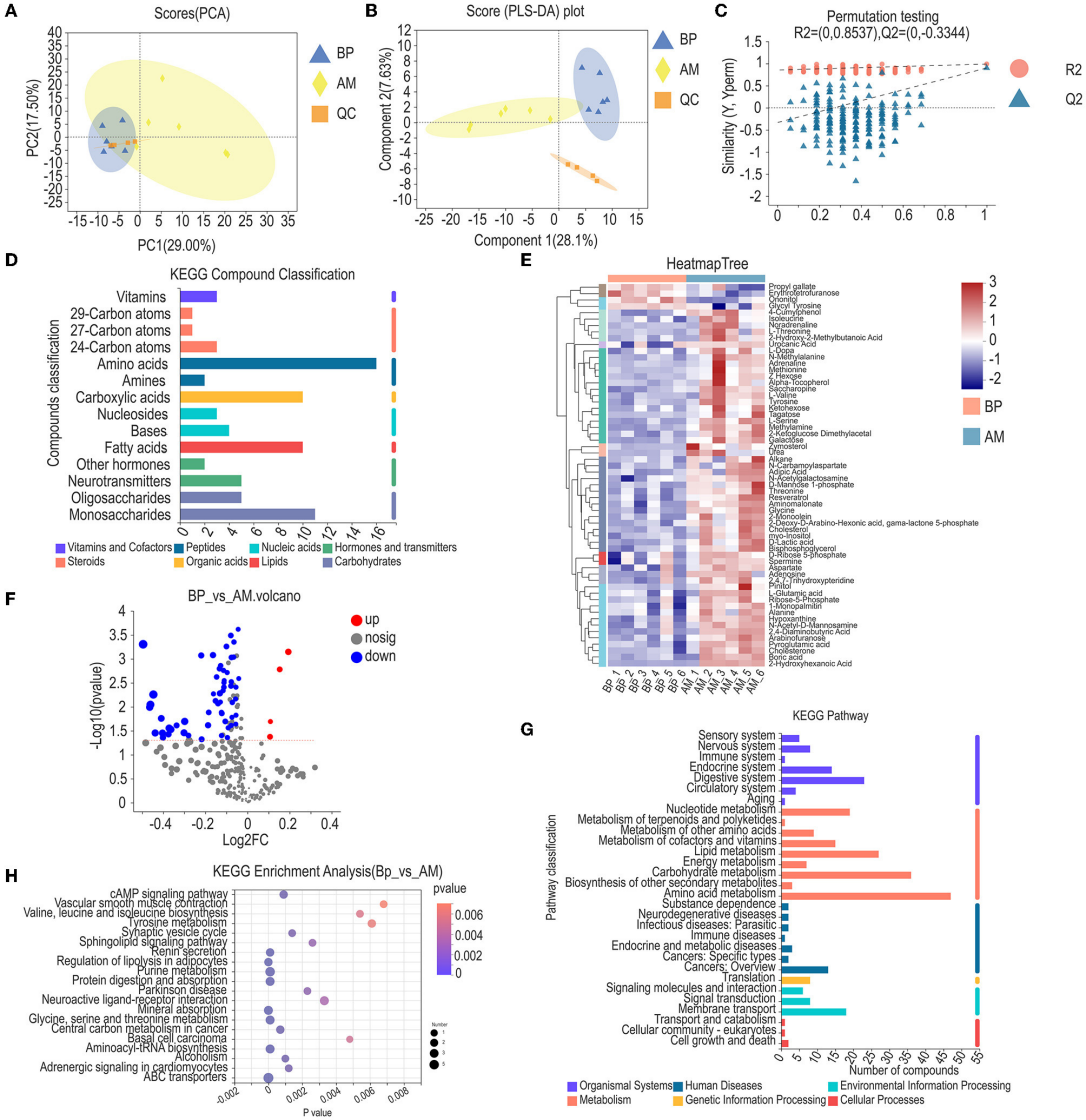
Metabolic spectrum analysis of longest dorsal muscle in meat rabbit. **(A)** PCA, **(B)** PLS-DA, **(C)** Permutation testing, **(D)** KEGG compound classification, **(E)** Heatmap tree, **(F)** Volcano diagram of differential metabolites, **(G)** KEGG pathway, and **(H)** KEGG enrichment analysis. Beet pulp treatment (BP), alfalfa meal treatment (AM), and peanut vine treatment (PV).

Through the analysis of apparent performance and microbiota, we found that the microbiome of AM treatment was similar to PV treatment, but the apparent performance of AM was better than that of PV. Furthermore, AM and BP were used to analyze muscle metabolism (Figure 6A). The PCoA for muscle metabolism in the BP and AM treatments found that the scores of PC1 and PC2 coordinates are 29.00 and 17.50%, respectively. BP and AM processing treatments can be divided (Figure 6B). PLS–DA analysis is required to detect the quality of metabolome models. The partial least square regression model was used to establish the relationship between metabolite expression and sample type (different test treatments) to achieve the prediction of sample type. Model evaluation parameters are (R2, Q2), when R2 and Q2 are closer to 1, indicating that the model is more stable and reliable, As shown, BP and AM treatments PLS–DA were 28.1% and 7.63% in one and two dimensions, respectively. Among them, R2Y = 0.8537, Q2y = 0.3344, indicating that the model between metabolite expression and experimental treatment is stable and reliable, and the predictive effect is good (Figure 6C).

The identified metabolites were functionally annotated through the KEGG database. The annotated metabolites were mainly concentrated in related substances such as amino acids, monosaccharides, fatty acids, carboxylic acids, and neurotransmitters (Figure 6D). The differential metabolites treated by BP and AM were analyzed by hierarchical cluster analysis, and a total of 60 differential metabolites were found (Figure 6E). The volcanic map of differential metabolites was obtained by multiple analysis and *t*-test. Fold change (FC) was obtained by multiple analysis of variance, and *p* was obtained by *t*-test. A total of 60 differential ions in the two treatments were identified under the conditions of VIP ≥ 1, fold change ≥ 1, and *P* < 0.05. The difference analysis showed that compared with AM treatment, 56 differential ions in BP treatment were significantly down-regulated and 4 differential ions were significantly up-regulated (Figure 6F). Furthermore, the identified metabolites are annotated through the KEGG database, as shown in Figure 6, the identified metabolites are classified and annotated. The annotated metabolites are mainly concentrated in the processes of amino acid metabolism, carbohydrate metabolism, digestive system, nucleoside metabolism, endocrine system, lipid metabolism, and others (Figure 6G). KEGG database was given to analyze the metabolic pathway enrichment of differential metabolites. The metabolic pathway with *p* < 0.05 is the metabolic pathway with significant enrichment of differential metabolites, as shown in the figure: a total of 20 differential metabolic pathways were annotated under BP and AM treatments. The main differential metabolic pathways are cAMP signaling pathway, glycine, serine, and threonine metabolism, regulation of lipolysis in adipocytes, Tyrosine metabolism, Valine, leucine, and isoleucine biosynthesis, and Sphingolipid signaling pathway (Figure 6H).

### Correlation Analysis Between gut Microbiota and Metabolic Spectrum of Longest Dorsal Muscle

**Figure 7:**
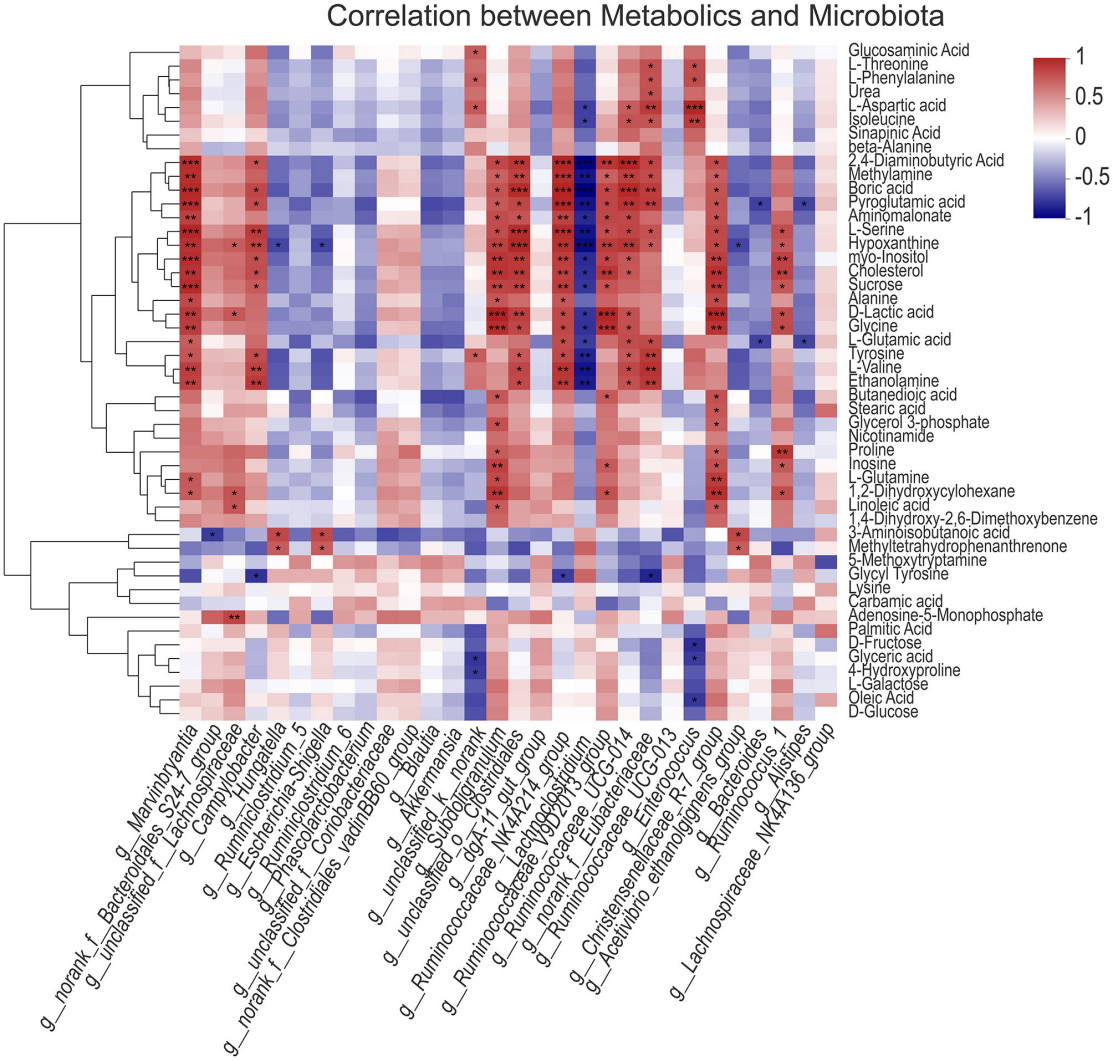
Correlation analysis between gut microbiota and metabolic spectrum of longissimus dorsi muscle. The asterisks symbol indicates significant correlation (*0.01 < *P* ≤ 0.05, **0.001 < *P* ≤ 0.01, ***P ≤ 0.001).

Further correlation analysis of gut microbiota and main metabolites showed that *Marvinbryantia, Campylobacter, subdoligranulum, unclassified_ o_ Clostridiales, Ruminococcaceae_ NK4A214_ group, Ruminococcaceae_ V9D2013_ group, G_ Ruminococcaceae_ UCG-014, norank_ f_ Eubacteriaceae, Christensenellaceae_ R-7_ group*, and other bacteria were significantly positively correlated with amino acids and carbohydrates, such as 2,4-diaminobutyric acid, pyroglutamic acid, aminomalonate, L-serine, sucrose, alanine, D-lactic acid, glycine, L-glutamic acid, tyrosine, L-valine, and other metabolites, while lachnoclostridium was significantly negatively correlated with them; *Hungatella, Escherichia Shigella*, and other bacteria are negatively correlated with some amino acid metabolites, indicating that there is a certain correlation between gut microbiota and muscle metabolites (Figure 7).

## Discussion

The rabbit is a herbivorous monogastric animal. Fiber is the main component of their diet. A large number of studies have shown that dietary fiber plays an important role in the growth of meat rabbits. Fiber and energy play an important role in diet composition in meat rabbits (1, 2, 8). Maintaining proper fiber and energy levels can effectively maintain the health of meat rabbits and take into account the optimum growth rate (22). Because of the wide sources, variety, and complex chemical structure of the dietary fiber, the effects of different dietary fibers vary greatly (21). Therefore, it is very important to select suitable dietary fiber types to satisfy the energy and fiber levels of meat rabbits. In this study, we found that dietary fiber type affects the average daily gain and nutrient digestibility of meat rabbits. In particular, the apparent digestibility of AM, average daily feed intake, average daily weight gain, dry matter, and neutral detergent fiber increased significantly. In addition, AM treatment significantly increased premortem live body weight and the half and full evisceration rate. At the same time, the apparent performance of PV treatment is also higher than that of BP. From the perspective of fiber fermentability, the fibers in alfalfa meal and peanut seedling powder mainly contain insoluble fibers, and the fibers in beet meal mainly contain soluble fibers. Previous studies have shown that adding high soluble fiber to the diet of meat rabbits will increase the relative weight of cecal contents and residence time in the cecum, affect their nutrient digestibility, reduce feed intake and daily gain, and finally affect their slaughter performance (23). The development of the intestine is directly related to the growth and development of meat rabbits. The small intestine is the main part for animals to digest and absorb food. Ensuring the normal operation of the structure and function of the small intestine is the basis for nutrients to be fully absorbed and utilized in the body (24). Previous studies have found that different fiber components in the diet affect the intestinal villus height and muscle layer thickness of jejunum, as well as the crypt depth of duodenum and ileum (25). Similarly, Gómez-Conde et al. found that dietary fiber could significantly affect the intestinal villus height of the duodenum and the ratio of villus height to crypt depth (26). In the current study, we found that AM and PV treatments had beneficial effects on villus height and crypt depth of the small intestine. It is suggested that the addition of AM and PV in the diet is more conducive to promoting the intestinal development of meat rabbits, and then improving the growth performance and slaughter performance of meat rabbits.

There is a reciprocal symbiotic relationship between gut microbiota and animals (27). Compared with other monogastric animals, the intestinal structure of rabbits is relatively unique. Its cecum volume can reach approximately 49% of the total volume of all gastrointestinal tract, which is the largest in monogastric animals (7). The cecum is the main fermentation organ. The front-end microbiota in the gastrointestinal tract of meat rabbits are less colonized and the chyme circulation speed is fast. The dietary fiber in the digestive tract before entering into the cecum of meat rabbits is not digested and utilized (28). Therefore, this study detected the cecum of meat rabbits and found that the dominant bacteria treated with AM were *Enterococcus, norank_ f__ Clostridiales_ vadinBB60_ group, norank_ f_ Bacteroidales_ S24-7_ group, Christensenellaceae_ R-7_ group*, and *Ruminococcaceae_ NK4A214_ group*. The dominant bacteria in PV treatment are *Enterococcus, norank_ f_ Eubacteriaceae, Ruminococcaceae_ NK4A214_ group, Christensenellaceae_ R-7_ group*, and *norank_ f_ Clostridiales_ vadinBB60_ group*. The dominant bacteria in BP treatment are *Escherichia Shigella, Bacteroides*, and *norank_ f__ Bacteroidales_ S24-7_ group* and *norank_ f_ Clostridiales_ vadinBB60_ group*. Interestingly, the microbiota of AM and PV treatments were relatively similar, which may be due to their similar fiber types, mainly containing insoluble fibers. It is reported that *Enterococcus faecalis* found in the rumen of Tibetan Yak (*Bos grunniens*) can produce cellulose (29). *Christensenellaceae_ R-7_ group* bacteria are plant fiber–degrading bacteria and produce short-chain fatty acids (29). *norank_ f_ Bacteroidales_ S24-7_ group* can significantly alleviate inflammation and inhibit harmful bacteria (30). Dietary fiber not only regulates the composition and function of gut microbiota, but can also regulate the composition of microbial metabolites such as SCFAs, improve intestinal health and body metabolism, and animal behavior (10, 11). Previous studies have found that high dietary fiber can enrich 15 SCFAs-producing bacteria in the intestine to produce SCFAs, improve the intestinal environment, increase the butyrate concentration, competitively inhibit other “harmful bacteria,” and reduce the production of harmful metabolites to build a healthier intestine (31). In this study, AM- and PV-treated meat rabbits are rich in *norank_ f_ Eubacteriaceae, Ruminococcaceae_ NK4A214_ group, Ruminococcaceae_ V9D2013_ group*, and *Ruminococcaceae_ Ucg-014* as compared to BP-treated meat rabbits which account for a higher relative abundance of *Bacteroides, lachnospiraceae*, and *Escherichia Shigella*. *Escherichia coli* is a conditional pathogen. When meat rabbits encounter an adverse environment or unreasonable ration, then *E. coli* feel the signal of the environment and respond to the changes of the environment in the form of secreting some toxins, destabilizing the cecal micro-ecosystem and hence causing diarrhea in meat rabbits (32). Beet meal mainly contains soluble fiber. Previous studies have found that a high soluble fiber in the meat rabbit diet will increase the viscosity of chyme, hinder the digestion and absorption of chyme, increase the relative weight of cecal contents and the residence time of chyme in the cecum, and then accumulate toxins in the cecum, resulting in environmental disorder in the cecum and abnormal growth of *E. coli*. (33). Many studies have confirmed that the metabolism in the cecum of meat rabbits is similar to that in the rumen, and there are cocci similar to the rumen in the cecum of meat rabbits (34). Ruminococcaceae can degrade cellulose and hemicellulose in feed, produce butyrate by fermenting complex non-digestible polysaccharides, and plays a pivotal role in the maintenance of intestinal health (35). This study, may explain the significant increase of butyric acid content in AM- and PV-treated meat rabbits. It is suggested that the changes of cecal microbiota and their metabolites mediated by insoluble dietary fiber may play an important role in maintaining gut microbiota balance and ensuring intestinal health.

Dietary fiber can regulate the composition and diversity of gut microbiota. gut microbiota can also affect the metabolism of liver, fat, and muscle tissue, which may further affect the network of nutrient intake and energy metabolism of the host (36). Metabonomics is widely used to study the effects of changes in animal nutrition, environment, genes, and gut microbiota ecological environment on the response pathway of the metabolic system (37). In this study, the non-targeted metabonomics of longissimus dorsi muscle of growing meat rabbits was analyzed by GC–MS. through PLS–DA modeling analysis, it was found that there were significant differences between the groups, indicating that different fiber sources can significantly affect muscle metabolism. The main metabolites identified in this study are concentrated in compounds with biological effects (amino acids, monosaccharides, fatty acids, carboxylic acids, neurotransmitters, etc.). On the basis of level 1 and level 2, four up-regulated differential metabolites and 56 down-regulated metabolites were identified, respectively. The identified metabolites were functionally annotated through the KEGG database. The main biochemical metabolic pathways and signal transduction pathways involved by these metabolites include amino acid metabolism, carbohydrate metabolism, digestive system, nucleoside metabolism, Endocrine system, and lipid metabolism. Through the analysis of differential metabolites and differential metabolic pathways, the metabolic pathways that produce significant differences are cAMP signaling pathway, glycine, serine, and threonine metabolism, regulation of lipolysis in adipocytes, tyrosine metabolism, valine, leucine, and isoleucine biosynthesis, and sphingolipid signaling pathway. Previous studies have shown that cAMP plays an important role in the treatment of cardiovascular diseases, skin diseases, and cancer. It can improve the body's immunity, enhance the body's hematopoietic function, prevent cancer, improve cardiovascular function, protect liver function, prevent allergic diseases, and improve brain function. It plays a vital role in some physiological activities in cells (38, 39). Verardo et al. found that the cAMP pathway has the potential to control muscle cell homeostasis and meat quality based on the SNP association pathway and transcription factor analysis (39).

**Figure 8:**
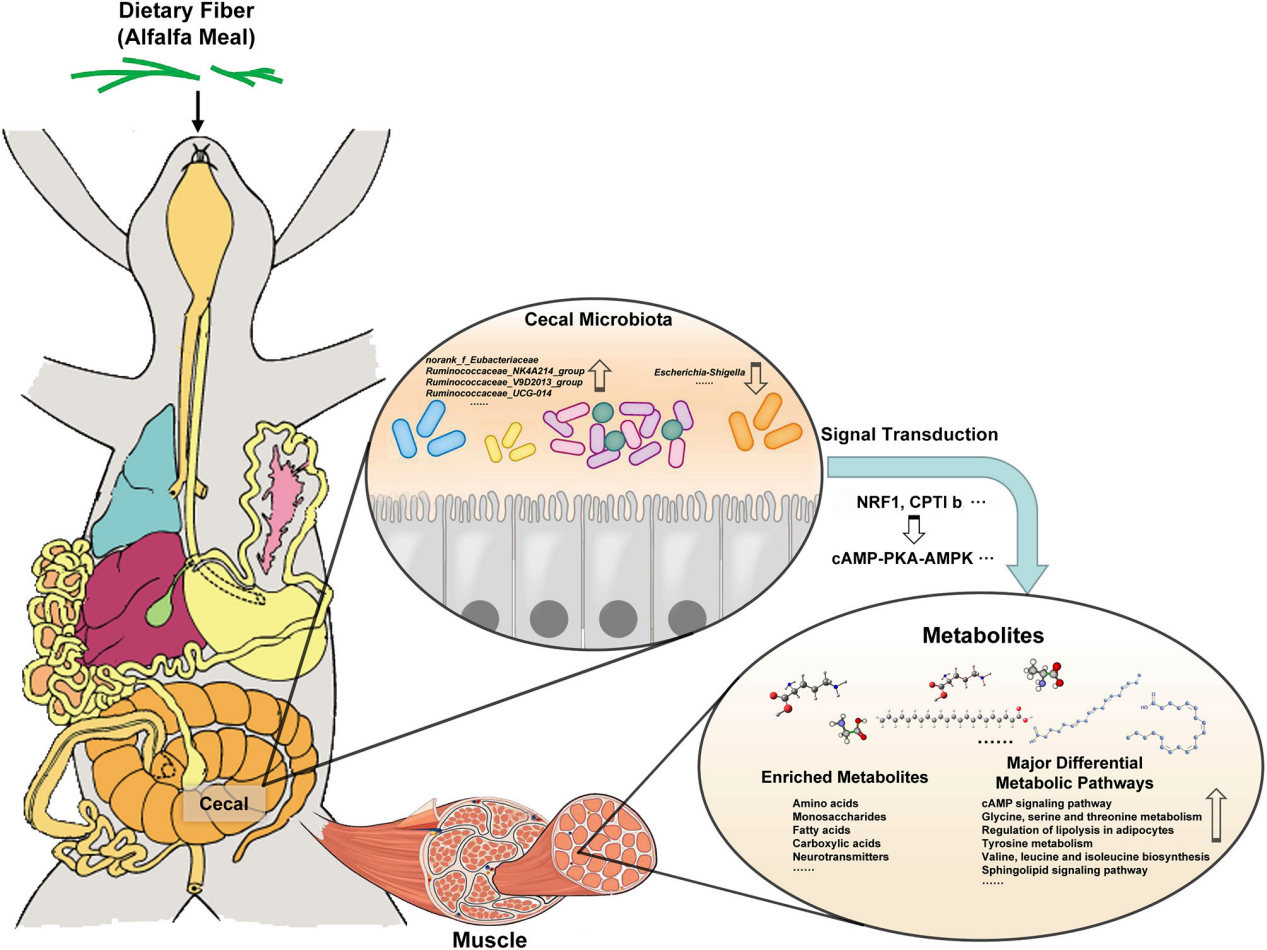
The addition of AM in diet may affect muscle metabolism by changing its gut microbiota, and then changing the flavor and quality of muscle.

Studies have shown that cAMP–PKA–AMPK signaling pathway can regulate AMPK activity and mitochondrial energy metabolism (19). It was found that in its signal pathway, the increased expression of NRF1 mRNA was directly proportional to oxygen content, which induced mitochondrial biosynthesis and thus incorporated in strengthening the level of oxidative phosphorylation. (40). CPTlb is located in the outer membrane of mitochondria, which promotes the combination of fatty acyl coenzyme A and carnitine to produce fatty acyl carnitine. Under the action of transferase, fatty acyl carnitine is transferred to the inner membrane of mitochondria and then initiates β-oxidation which provides energy for the body, promotes fat metabolism, and reduces fat deposition (20). It shows that AM treatment can promote mitochondrial biosynthesis and energy metabolism. In addition, it was found that the type and content of amino acids were related to muscle quality and flavor. The content of essential amino acids determines the quality of muscle protein and the main flavor amino acids such as glutamate, glycine, aspartic acid, and alanine provide the material basis for muscle flavor (41, 42). This study found that AM treatment significantly improved the metabolic pathways of amino acids such as glycine, serine, and threonine metabolism, tyrosine metabolism, valine, leucine, and isoleucine biosynthesis. These results showed that AM treatment was beneficial to the accumulation of amino acids and essential amino acids in rabbit muscle and improved the flavor and quality of rabbit meat. Interestingly, through the correlation analysis between the cecal microbiota of meat rabbits and the main metabolites in muscle, it was found that there was a significant correlation between rumen cocci and amino acid metabolites in the muscle. It further shows that there is a correlation between gut microbiota and muscle metabolites, which provides a theoretical basis for the gut muscle axis. It is suggested that the addition of AM in diet may affect the flavor and quality of muscle by modulating its gut microbiota (Figure 8). However, further studies are needed to clarify the specific mechanism of the interaction between gut microbiota and muscle metabolites.

## Conclusions

Taken together, our results indicate that AM treatment may improve the growth performance and other apparent parameters by changing gut microbiota, and then affect the quality of rabbit meat. Microbial composition showed that AM treatment and PV treatment significantly improved the diversity and richness of gut microbiota in rabbits, and their gut microbiota was more similar. In addition, they promoted the relative abundance of cellulolytic bacteria in the gut, promoted fiber fermentation, reduced the relative abundance of pro-inflammatory bacteria, and maintained the balance of intestinal microecology. In addition, the metabonomics analysis screened some differential metabolites, which were related to meat energy metabolism and amino acid flavor, and mainly concentrated in the cAMP signaling pathway, Glycine, serine, and threonine metabolism, Regulation of lipolysis in adipocytes, Tyrosine metabolism, Valine, leucine, and isoleucine biosynthesis, and other metabolic pathways. Through the correlation analysis between the meat rabbit microbial community and main metabolites in muscle, it was found that there was a significant correlation between the rumenococci in the cecum and amino acid metabolites in the muscle. This may help us regulate gut microbiota and promote body development through dietary fiber, instead of using antibiotics in large quantities, which may reduce bacterial drug resistance and improve meat product safety.

## Data Availability Statement

The datasets presented in this study can be found in online repositories. The names of the repository/repositories and accession number(s) can be found below: https://www.ncbi.nlm.nih.gov/, SRP346579.

## Ethics Statement

The animal study was reviewed and approved by Animal Ethics Committee of Henan Agricultural University. Written informed consent was obtained from the owners for the participation of their animals in this study.

## Author Contributions

BL and YC performed experiments and analyzed data. XZ participated in the data collection. QA assisted with animal experimentation. DL, SM, ZW, and CW provided advice in design and performance of experiments. BL wrote the manuscript draft. YS supervised the study. All authors read and approved the final manuscript.

## Funding

Financial support for this research was provided by the China Agriculture Research System of MOF and MARA (CARS-34).

## Conflict of Interest

The authors declare that the research was conducted in the absence of any commercial or financial relationships that could be construed as a potential conflict of interest.

## Publisher's Note

All claims expressed in this article are solely those of the authors and do not necessarily represent those of their affiliated organizations, or those of the publisher, the editors and the reviewers. Any product that may be evaluated in this article, or claim that may be made by its manufacturer, is not guaranteed or endorsed by the publisher.
