# Supplementary material for: Gut Microbiota Modulate Rabbit Meat Quality in Response to Dietary Fiber
Source: Front Nutr. 2022 Mar 22;9:849429. doi: 10.3389/fnut.2022.849429 (PMC8982513; doi:10.3389/fnut.2022.849429)
Supplement: Supplementary file 1 [file Table_1.DOCX]

Supplementary Material

# Supplementary Table

Table S1 Composition and nutritional level of experimental diet (Air-drybasis) g/kg

| Items | Beet pulp | Alfalfa meal | Peanut vine |
| --- | --- | --- | --- |
| Corn | 4 | 10.8 | 16.17 |
| Wheat middling | 4.3 | 7.03 | 5 |
| Soybean meal | 21.8 | 10.8 | 18.98 |
| Wheat bran | 23.5 | 23.87 | 15 |
| Peanut vine | 0 | 0 | 41.85 |
| Alfalfa meal | 0 | 44.5 | 0 |
| Beet pulp | 43.8 | 0 | 0 |
| CaHPO4 | 1.5 | 1.5 | 1.5 |
| Nacl | 0.1 | 0.5 | 0.5 |
| Premix | 1 | 1 | 1 |
| Total | 100 | 100 | 100 |
| Nutrient levels |  |  |  |
| DE ^a^（MJ/Kg） | 10.91 | 10.85 | 10.86 |
| CP ^a^（%） | 18.29 | 18.11 | 18.4 |
| NDF ^a^（%） | 31.09 | 31.55 | 31.56 |
| ADF ^a^（%） | 13.57 | 17.56 | 20.98 |
| EE ^a^（%） | 2.46 | 2.78 | 2.77 |
| CF ^a^（%） | 10.11 | 13.35 | 11.33 |
| Ca ^a^ | 0.6 | 0.59 | 0.61 |
| P ^a^ | 0.65 | 0.67 | 0.66 |

^a^ DE: Digestion energy, CP: Crude protein, CF: Crude fiber, NDF: Neutral detergent fibre, ADF: Acid Detergent Fiber, Ca: Calcium, P: Phosphorus

Table S2 The primer sequence of the studied genes

| Gene | Forward primer (5′------ 3′) | Reverse primer (5′------ 3′) |
| --- | --- | --- |
| *NRF1* | CATTGTTCTCTGTATCTCACCCTCC | TGTACTTACGCACCACATTCTCC |
| *CPT1B* | ACCCAGACCAGTACCCCAATC | TGCTGGAGACGTGGAAGAAGAT |
| *GADPH* | AGAGCACCAGAGGAGGACG | TGGGATGGAAACTGTGAAGAG |
